# High glucose-induced hyperosmolarity contributes to COX-2 expression and angiogenesis: implications for diabetic retinopathy

**DOI:** 10.1186/s12933-016-0342-4

**Published:** 2016-01-29

**Authors:** Rosalinda Madonna, Gaia Giovannelli, Pamela Confalone, Francesca Vera Renna, Yong-Jian Geng, Raffaele De Caterina

**Affiliations:** Laboratory of Experimental Cardiology, Center of Excellence on Aging, Institute of Cardiology, “G. d’Annunzio” University, C/o Ospedale SS. Annunziata, Via dei Vestini, 31, 66013 Chieti, Italy; The University of Texas Health Science Center at Houston and the Texas Heart Institute, Houston, TX USA; Department of Neurosciences and Imaging, “G. d’Annunzio” University, Chieti, Italy

**Keywords:** Glucose, Diabetes, Hyperosmolarity, Cyclooxygenase-2, Angiogenesis, Microangiopathy, Diabetic retinopathy

## Abstract

**Background:**

We tested the hypothesis that glucose-induced hyperosmolarity, occurring in diabetic hyperglycemia, promotes retinal angiogenesis, and that interference with osmolarity signaling ameliorates excessive angiogenesis and retinopathy in vitro and in vivo.

**Methods and Results:**

We incubated human aortic (HAECs) and dermal microvascular endothelial cells (HMVECs) with glucose or mannitol for 24 h and tested them for protein levels and in vitro angiogenesis. We used the *Ins2 Akita* mice as a model of type 1 diabetes to test the in vivo relevance of in vitro observations. Compared to incubations with normal (5 mmol/L) glucose concentrations, cells exposed to both high glucose and high mannitol (at 30.5 or 50.5 mmol/L) increased expression of the water channel aquaporin-1 (AQP1) and cyclooxygenase (COX)-2. This was preceded by increased activity of the osmolarity-sensitive transcription factor Tonicity enhancer binding protein (TonEBP), and enhanced endothelial migration and tubulization in Matrigel, reverted by treatment with AQP1 and TonEBP siRNA. Retinas of *Ins2 Akita* mice showed increased levels of AQP1 and COX-2, as well as angiogenesis, all reverted by AQP1 siRNA intravitreal injections.

**Conclusions:**

Glucose-related hyperosmolarity seems to be able to promote angiogenesis and retinopathy through activation of TonEBP and possibly increasing expression of AQP1 and COX-2. Osmolarity signaling may be a target for therapy.

## Background

Hyperglycemia can cause diabetic micro- [1] and macrovascular complications [2] by triggering oxidative stress [3, 4], forming advanced glycation end products (AGEs) [4, 5], increasing the flux of glucose to sorbitol through the polyol and hexosamine pathways [3], activating protein kinase C (PKC) [6], and provoking inflammation [7] (reviewed in [8]). Diabetic microvascular disease is pathologically characterized by excessive vessel growth and increased vessel permeability [9, 10].

Hyperglycemia has been reported to increase the endothelial expression of the proinflammatory proteins intercellular adhesion molecule (ICAM)-1 and vascular cell adhesion molecule (VCAM)-1, and to decrease production of nitric oxide, all through activation of the water channel protein aquaporin (AQP)1 [11]. Hyperosmotic stress inevitably accompanies hyperglycemia, and may therefore contribute to some aspects of hyperglycemia-induced vascular injury.

Similarly to VCAM-1 and ICAM-1, cyclooxygenase (COX)-2 participates in inflammation [12] and angiogenesis [13, 14]. Induced by cytokines, mitogens and endotoxin [12], COX-2 regulates the expression and activity of matrix metalloproteinase (MMP)-9 [15], which degrades extracellular matrix and promotes endothelial cell migration and vessel sprouting [16]. Endothelial COX-2 has been implicated in angiogenesis (mostly in the context of tumorigenesis, reviewed in [17]), as well as in proliferative diabetic retinopathy [18], characterized by excessive angiogenesis [19–21]. COX-2 is expressed in human monocytes and macrophages, as well as human endothelial cells [22] exposed to high glucose [7].

Against this background, we therefore examined whether hyperosmotic stress, at levels attainable in diabetes, induces COX-2 expression in human micro- and macrovascular endothelial cells. We then analyzed the functional impact of hyperosmotic stress and COX-2 expression on angiogenesis and explored underlying mechanisms, particularly with regard to the involvement of water channels and associated signaling molecules. Finally, we investigated the in vivo implications of such findings for diabetic retinopathy in a mouse model.

## Methods

### Materials

d-glucose, D-mannitol and l-glucose (these two latter devoid of metabolic activities and used as a purely hyperosmolar controls), saccharose, and sodium chloride, were purchased from Sigma (St. Louis, MO, USA). The selective COX-2 inhibitor NS-398 was purchased from Calbiochem (Gibbstown, NJ, USA).

### Animal care

Male C57BL/6 mice (body weight: 15 ± 4 g, age: 1 year; n = 21) and male D2.B6-*Ins2 Akita* diabetic mice, a model of type 1 diabetes developing retinal angiogenesis [23, 24] (body weight: 11 ± 2 g, n = 21) were purchased from The Jackson Laboratories (Bar Harbor, ME, USA). All procedures were approved by our Institutional Ethics Committee for Animal Research. Investigations conformed to the Principles of Laboratory Animal Care formulated by the National Society for Medical Research and the Guide for the Care and Use of Laboratory Animals [NIH Publication 86–23, 1985 revision].

### Cell cultures

Subconfluent human aortic endothelial cells (HAECs) and human microvascular endothelial cells (HMVECs) at passage 3 were synchronized by starvation in Endothelial Basal Medium (EBM, Clonetics, Baltimore, MD, USA) for 24 h, then incubated with control d-glucose concentration (5.5 mmol/L, control and 285 mOsm/L), high glucose (HG: 30.5 mmol/L and 385 mOsm/L or 50.5 mmol/L and 460 mOsm/L), high mannitol (HM: 5.5 mmol/L glucose + 25 mmol/L and 385 mOsm/L or 45 mmol/L mannitol and 385 mOsm/L), for 24 or 72 h. In some experiments, cells were also incubated with l-glucose at equimolar concentrations, saccharose or sodium chloride, as further osmotic controls, in the presence or absence of 1–10 μg/mL NS-398.

Cell viability after treatments was assessed by cell morphology and cell count at phase-contrast microscopy, Trypan blue exclusion, and total protein content.

### Immunoblotting

Total proteins from retinas of wild-type C57BL/6 male mice and sex/age matched D2.B6-*Ins2 Akita* diabetic mice, or from HMVECs and HAECs were isolated, electroblotted, and incubated with the following primary and secondary antibodies: (1) goat polyclonal anti-COX-2 (cat. N° sc-1747, dilution 1:500, Santa Cruz Biotechnologies, Santa Cruz, CA USA), secondary antibody mouse anti-goat IgG-HRP (cat. N° sc-2354, dilution 1:5000, Santa Cruz); (2) mouse monoclonal anti-AQP1 (cat. N° sc-55466, dilution 1:600, Santa Cruz), secondary antibody goat anti-mouse IgG-HRP (cat. N° sc-2005, dilution 1:5000, Santa Cruz); (3) mouse monoclonal anti-β-actin (cat. N° A5441, dilution 1:5000, Sigma, St. Louis, MO, USA), secondary antibody goat anti-mouse IgG-HRP (cat. N° sc-2005, dilution 1:5000, Santa Cruz); (4) rabbit polyclonal anti-TonEBP (cat. N° sc-13035, dilution 1:500, Santa Cruz), secondary antibody goat anti-rabbit IgG-HRP (cat. N° sc-12304, dilution 1:10000, Santa Cruz), as previously described [25].

### Transfection of AQP1 and tonicity enhancer binding protein (TonEBP)/nuclear factor of activated T cells (NFAT)5 siRNA

A pool of three different small interfering RNA (siRNA) oligonucleotides to each target (AQP1 and TonEBP/NFAT5), as well as scrambled control siRNA were incubated with cells (2 × 10^5^/well) in transfection medium, as previously described [26]. After 24 h, fresh medium was added with or without HG (30.5 or 50.5 mmol/L), or HM (5.5 mmol/L glucose + 25 or 45 mmol/L mannitol), incubated for an additional 24 h, then subjected to Western analysis of COX-2, AQP1, TonEBP/NFAT5 and β-actin.

### Tube formation assays

Tube formation assays were performed as previously described [26]. Before the assay, cells were incubated in low serum (2.5 %) endothelial basal medium (EBM, BD Biosciences) without endothelial growth factors, with concentrations of control glucose (5.5 mmol/L, control), high glucose (30.5 or 50.5 mmol/l), high mannitol (5.5 mmol/L glucose + 25 or 45 mmol/L mannitol) for 24 h, in the presence or absence of the selective cyclooxygenase(COX)-2 inhibitor NS-398 at 1–10 μg/mL. In the assay testing the effect of siRNA to aquaporin (AQP)1 or TonEBP, cells were pretreated with high glucose or high mannitol for 24 h. At the end of incubations, cells were gently detached with 0.25 % trypsin–EDTA; trypsinized cells were then collected, counted and plated on Matrigel (at a density of 2 × 10^5^ cells/50 μL Matrigel) for a further 16 h, in the presence or absence of control glucose, high glucose, high mannitol, with or without NS-398, or siRNA to AQP1 or TonEBP.

### Cell migration assay

Cell migration was measured using the CytoSelect™ cell migration assay (Cell Biolabs, San Diego, CA, USA), according to the manufacturer’s instructions. Following a 24 h starvation period in 2 % fetal calf serum (FCS)/endothelial cell growth medium (EGM), cells were resuspended at 4 × 10^5^ cells/mL in EGM and then placed in a separate well of a 96-well feeder tray containing 150 μL of migration media (EGM with control glucose concentration (5.5 mmol/L, control), high glucose (HG 30.5 mmol/L) or high mannitol (HM HM, 5.5 mmol/L glucose + 25 mmol/L mannitol)). One hundred and fifty microliters of assay controls, consisting of EGM supplemented with 10 % FCS (positive control) and basal EGM (negative control), were also tested in triplicate.

### Electrophoretic mobility gel shift assay

Nuclear proteins were extracted from treated or untreated endothelial cells and subjected to electrophoretic mobility gel shift assay, as previously described [25]. Double-stranded synthetic oligonucleotides of the nuclear factor (NF)-κB motif (5′-AGT TGA GGG GAC TTT CCC AGG C-3′ and 5′-CCT GGG AAA GTC CCC TCA ACT-3′) or of the Tonicity enhancer element (5′-GGT TTC TCC ACC TTT TCC GCT G-3′) were labeled with [γ-^32^P]ATP.

### Immunoblotting of TonEBP

15 µg of nuclear extracts used for EMSA and of cytosolic extracts were electroblotted and incubated with either a primary rabbit polyclonal anti-TonEBP (cat. N° sc-13035, dilution 1:500, Santa Cruz), secondary antibody goat anti-rabbit IgG-HRP (cat. N° sc-12304, dilution 1:10,000, Santa Cruz); or an anti-lamin B antibody (Santa Cruz), as previously described [25].

### Preparation of transit TKO-siRNA complexes and intravitreal injections of non-viral siRNA

Non-viral siRNA delivery into the mouse retina in vivo was done using a modified version of published methods [27]. For monitoring transfection efficacy we used scrambled siRNA conjugated with a Cy3 fluorochrome provided by Mirus (Mirus Bio, Madison, WI, USA). Accordingly, 1.33 μL (25 nM) of Cy3-conjugated scrambled siRNA or Cy3-conjugated AQP1-siRNA (Ambion) were combined with 0.5 μL of Transit-TKO (Mirus) in a final volume 10 μL sterile H_2_O, and incubated for 30 min at room temperature. Wild-type C57BL/6 mice and age-sex matched D2.B6-*Ins2 Akita* diabetic mice were anesthetized by intraperitoneal injection of ketanest/xylazine (ketanest, 100–125 mg/kg; xylazine, 10–12.5 mg/kg). Intravitreal injections of phosphate-buffered saline (PBS, sham group, N = 7 wild-type C57BL/6 mice and N = 7 D2.B6-*Ins2 Akita* mice), or scrambled-siRNA (N = 7 wild-type C57BL/6 mice and N = 7 D2.B6-*Ins2 Akita* mice), or AQP1-siRNA (N = 7 wild-type C57BL/6 mice and N = 7 D2.B6-*Ins2 Akita* mice) were performed under a dissecting microscope with a 32 gauge needle attached to a 5 μL glass syringe (Hamilton, Reno, NV, USA). The needle was positioned 1 mm posterior to the limbus, where 5 μL of the solution were slowly (in 3–5 s) injected into the vitreous chamber of the eye. Injections were repeated after 72 h and 1 week from the first injection.

### Tissue preparation and histological evaluation of vessel density

After the induction of anesthesia, retinas from D2.B6-*Ins2 Akita* diabetic mice and age/sex-matched C57/BL6 control mice were removed, embedded in optimal-cutting-temperature (OCT) medium, frozen on dry ice, and stored at −70 °C until sectioning. For immunofluorescence staining, 5 μm thick sagittal and frontal cryosections were obtained by using a sliding cryotome. These sections were permeabilized, blocked for 30 min in PBS containing 1 % bovine serum albumin, and incubated for 1 h at 4 °C with purified primary antibody anti-CD31 or Cy3-conjugated anti-α-smooth muscle actin (ASMA). A non-immune IgG (Becton, Dickinson and Co., Franklin Lakes, New Jersey, USA) was used as the isotype control. After washing with PBS, retinal sections stained with anti-CD31 antibody were incubated with phycoerythrin (PE)-conjugated secondary antibody, washed, mounted, and viewed with an immunofluorescence microscope. Sections were counterstained with 4′, 6-diamidino-2-phenylindole (DAPI) to identify nuclei. For immunohistochemistry, retinas were embedded in five separate paraffin blocks. Paraffin-embedded limb sections were deparaffinized, rehydrated, and stained with a primary antibody against CD31. Sections were counterstained with hematoxylin and eosin for structural analysis and to identify nuclei. Arterioles and capillaries were counted in a blinded manner in five randomly selected fields at 10× magnification. Vascular images were captured by using an inverted light microscope (Olympus IX71), and analyzed by using the Image-J software (Media Cybernetics, Rockville, MD, USA). Vessel density was expressed as the number of arterioles or capillaries per mm^2^.

### Statistical analysis

Two-group comparisons were performed by the Student’s t test for unpaired values. Comparisons of means of ≥3 groups were performed by analysis of variance (ANOVA), and the existence of individual differences, in case of significant F-values at ANOVA, tested by Scheffé’s multiple contrasts.

## Results

### High glucose induces COX-2 expression in human endothelial cells through a hyperosmolar mechanism

**Fig. 1:**
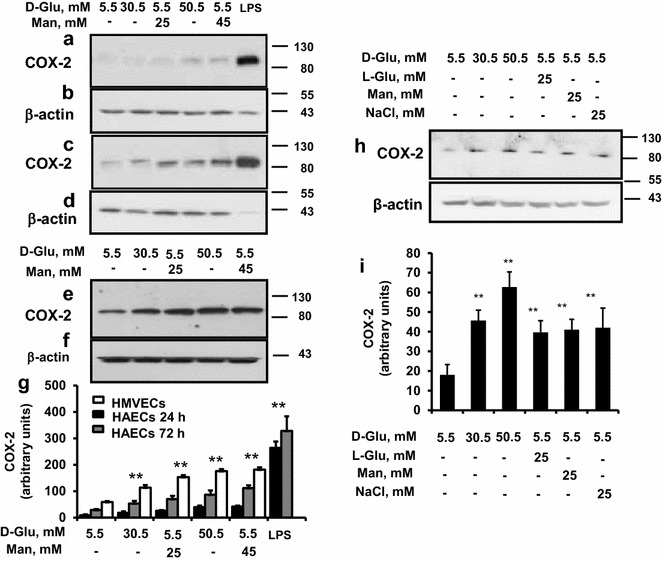
The effect of high glucose and high mannitol on COX-2 expression in endothelial cells. Immunoblotting with an antibody against COX-2 of proteins extracted from serum-starved subconfluent human aortic endothelial cells (HAECs) incubated with control D-glucose (5.5 mmol/L, control), high glucose (HG, 30.5 or 50.5 mmol/L), high mannitol (HM, 5.5 mmol/L glucose + 25 or 45 mmol/L mannitol) for 24 h (**a, b**) and for 3 days (**c, d**). After COX-2 analyses, proteins were stripped and restained with an anti-β-actin antibody. Lipopolysaccharide (LPS, 1 μg/mL) was used as positive control for COX-2 expression (**a, d**). **e, f** Immunoblotting with an antibody against COX-2 of proteins extracted from serum-starved subconfluent human microvascular endothelial cells (HMVECs) incubated with stimuli (12.5 and 25 mmol/L glucose or mannitol) for 3 days. Representative immunoblots are shown. The results of scanning densitometry (n = 3 independent experiments) are expressed as arbitrary units in* panel* **g**. *Columns* and *bars* represent the mean ± SD (*, P < 0.05 vs control cells; **, P < 0.01 vs control cells). Equal loading/equal protein transfers were verified by protein band detection with the anti-β-actin antibody. **h, i** The effect of high D-glucose, high l-glucose, high mannitol and NaCl on COX-2 expression in endothelial cells. Immunoblotting with an antibody against COX-2 of proteins extracted from serum-starved subconfluent human aortic endothelial cells (HAECs) incubated with control glucose concentration (5.5 mmol/L, control), high glucose (30.5 mmol/L), high mannitol (5.5 mmol/L glucose + 25 mmol/L mannitol), or sodium chloride (30.5 mmol/L), for 24 h. After COX-2 analyses, proteins were stripped and restained with an anti-β-actin antibody. Representative immunoblots from three different experiments are shown. **i** *Columns* and* bars* represent the mean ± SD of scanning densitometry of panel H (*, P < 0.05 vs control cells; **, P < 0.01 vs control cells)

Both short-term and longer-term exposure to high concentrations of glucose or mannitol, increased the expression of COX-2 protein, as shown by Western analysis (Fig. 1a–g). Similar results were obtained by incubating HAECs and HMVECs with l-glucose and NaCl at equimolar concentrations (30.5 mmol/L), as a further osmotic controls (Fig. 1h–i).

### AQP1 mediates high glucose-induced COX-2 expression in endothelial cells

**Fig. 2:**
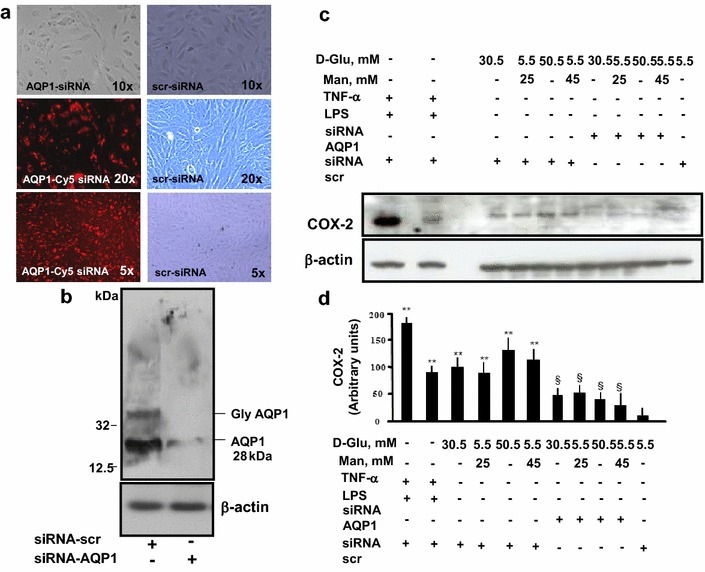
The effect of siRNA to AQP1 on COX-2 expression in endothelial cells exposed to high glucose and high mannitol. Serum-starved HAECs were transfected with Cy5-coniugated-siRNA to AQP1 or non-coniugated-siRNA to a scrambled sequence for 24 h and then treated with stimuli for 24 h. At the end of treatments, transfection efficiency was checked by observing the plates under fluorescence microscopy (**a**), while AQP1 gene knockdown efficiency was detected at the protein level by Western analysis (**b**). Protein expression for COX-2 was detected by Western analysis, with β-actin serving as a loading control (**c**). LPS (1 μg/mL) and tumor necrosis factor (TNF)-α (10 ng/mL) were here used as positive controls for the expression of COX-2. Representative blots (**c**) are shown. The results of scanning densitometry (n = 3 independent experiments) are expressed as arbitrary units in* panel* **d**, where *columns* and *bars* represent the mean ± SD (**, P < 0.01 mannitol- or glucose-treated vs control HAECs; §, P < 0.05 vs without AQP1-siRNA)

Since AQP1 regulates responses to hyperosmolarity in endothelial cells [28], we tested whether AQP1 is involved in hyperosmolarity-induced COX-2 expression. We used targeted disruption of AQP1 gene expression by siRNA technology, with 3 different siRNA species, to specifically block AQP1. AQP1 siRNA did not affect cell morphology or cell proliferation (Fig. 2a), but triggered a substantial (80 %) reduction in AQP1 protein expression (Fig. 2b). By contrast, serving as a sequence-unrelated control, scrambled siRNA did not exert any detectable effect. Proteins from AQP1 siRNA-transfected cells were then probed for COX-2 abundance. COX-2 expression in high glucose- and high mannitol-treated cells was substantially suppressed after transfection with siRNA against AQP1 (Fig. 2c–d). Taken together, these results showed that hyperosmolarity caused by exposure to high glucose or mannitol induces COX-2 expression through the activation of AQP1.

### High glucose promotes angiogenesis through hyperosmolarity-induced expression of COX-2 and its transcription factor TonEBP/NFAT5

**Fig. 3:**
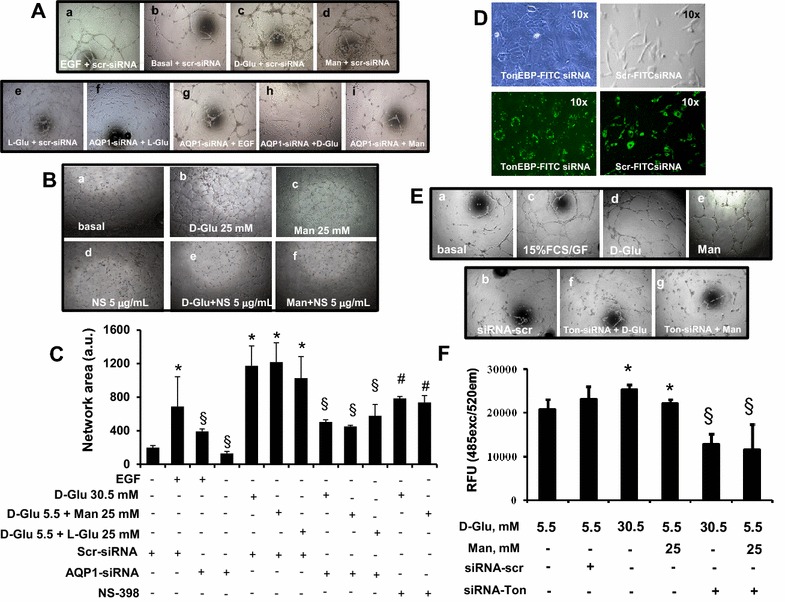
The involvement of AQP1, COX-2 and TonEBP/NFAT5 in glucose-induced in vitro angiogenic activity. **A, C, D** The effect of gene silencing to AQP1 or to TonEBP/NFAT5 on high glucose- and high mannitol-induced angiogenic activity of endothelial cells.** B** The effect of the COX-2 inhibitor NS-398 on high glucose- and high mannitol-induced angiogenic activity of endothelial cells.** E** The effect of TonEBP/NFAT5 gene silencing on high glucose- and high mannitol-induced endothelial cell migration. Serum-starved human aortic endothelial cells (HAECs) were transfected with Cy5-coniugated-siRNA to AQP1 or with FITC-coniugated-siRNA to TonEBP/NFTA5 and/or siRNA with scrambled sequence for 24 h (**D**), and then treated with stimuli for 24 h with high glucose or high mannitol. At the end of treatments, transfection efficiency was checked by observing the plates under fluorescence microscopy (**D**), while TonEBP/NFAT5 gene knockdown efficiency was detected at the protein level by Western analysis (**D**). At the end of incubations cells were enzymatically harvested, plated on Matrigel-coated 96 well-plates for the in vitro angiogenesis assay (**A–C, E**) or cell migration assay (**F**), and further incubated with stimuli for 24 h. In parallel experiments (**B**), serum starved HAECs were treated with high glucose or high mannitol (both at 25 mmol/L) in the presence or absence of 5 µg/mL NS-938 (NS) for 48 h and, after enzymatic detachment, plated on Matrigel-coated 96 well-plates, and further incubated with stimuli for 24 h. After incubation, images to document angiogenic activity were taken and analyzed as indicated in Methods.* Upper panels* (**A–E**): representative images.* Lower panels* (**C, F**): quantitation of angiogenic activity (network area, **C**) and for cell migration (**F**). *, P < 0.05 mannitol- or glucose-treated vs control HAECs; §, P < 0.05 vs without AQP1-siRNA or without TonEBP/NFAT-siRNA; # P < 0.05 vs without NS-938. RFU: relative fluorescence units

**Fig. 4:**
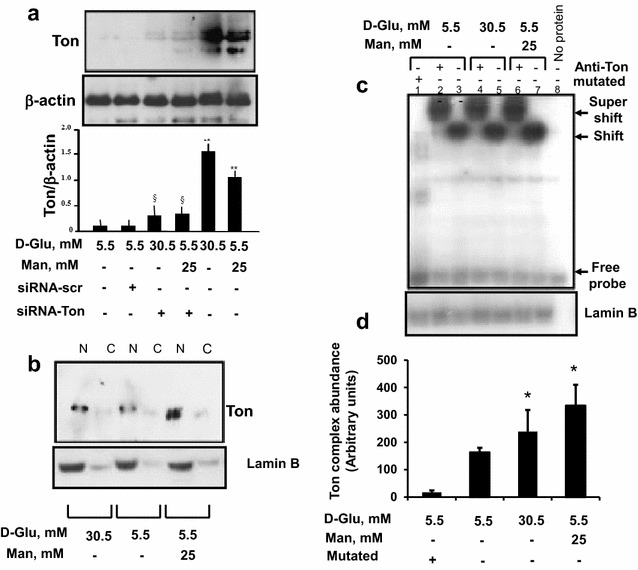
The effect of high glucose and high mannitol on TonEBP/NFAT5 expression and activity in endothelial cells. **a** Western analysis of TonEBP/NFAT5 in endothelial cells exposed to high glucose and high mannitol. Serum-starved HAECs were transfected with fluorescein (FITC)-coniugated-siRNA to TonEBP/NFAT5 or siRNA to scrambled sequence for 24 h, and then treated with stimuli for 24 h with high glucose or high mannitol. At the end of treatments, transfection efficiency was checked by observing the plates under fluorescence microscopy. Protein expression for TonEBP/NFAT was detected by Western analysis, with β-actin serving as loading control. Data represent for each condition the mean ± S.D. from three separate experiments. **, P < 0.01 mannitol- or glucose-treated *vs* control HAECs; §, P < 0.05 *vs* without TonEBP/NFAT-siRNA. **b, c, d** Recruitment of TonEBP/NFAT5 to TonE DNA binding sites in osmotically stressed human aortic endothelial cells. **b** Expression and distribution of TonEBP/NFAT5 in osmotically stressed HAECs for 1 h. Nuclear (N) and cytosolic (C) extracts were analyzed by Western blotting, using monoclonal anti-rabbit Ton antibody, with anti-lamin B serving as a loading control. **c** Electrophoretic mobility gel shift assay (*EMSA*), showing the effect of hypertonic stress on TonEBP/NFAT5 activation. EMSA was performed by mixing the ^32^P-oligonucleotide encoding for the TonEBP/NFAT5 binding probe with nuclear extracts from osmotically stressed HAECs for 1 h. Electrophoretic runs with nuclear protein extracts from unstimulated HAECs are shown in *lanes* 2 through 7. The run with a mutated DNA sequence is shown in *lane* 1. Results shown here are representative of three separate experiments. **d** Scanning densitometry of the EMSA gels, expressed as arbitrary units of optical density. Data represent for each condition the mean ± S.D. from three separate experiments. *, P < 0.01 mannitol- or glucose-treated vs control HAECs

**Fig. 5:**
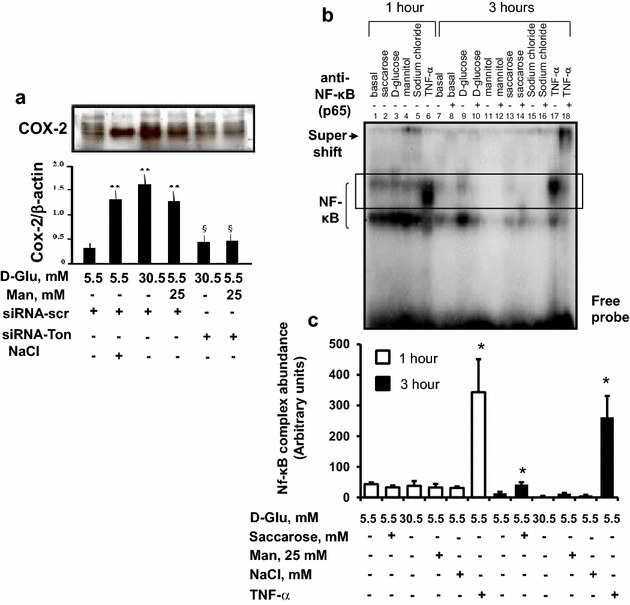
The involvement of TonEBP/NFAT5 in glucose-induced endothelial COX-2 expression. **a** Western analysis of the effect of siRNA to TonEBP/NFAT5 on COX-2 expression in endothelial cells exposed to high glucose and high mannitol. Serum-starved human aortic endothelial cells (HAECs) were transfected with fluorescein isothiocyanate (FITC)-coniugated-siRNA to TonEBP/NFAT5 or siRNA to scrambled sequence for 24 h, and then treated with stimuli for 24 h with high glucose, or high mannitol, or equimolar concentration of sodium chloride. At the end of treatments transfection efficiency was checked by observing the plates under fluorescence microscopy. Protein expressions for cyclooxygenase (COX)-2 was detected by Western analysis, with β-actin serving as loading control. Data represent for each condition the mean ± S.D. from three separate experiments. **, P < 0.01 mannitol- or glucose-treated *vs* control HAECs; §, P < 0.05 vs without TonEBP/NFAT-siRNA. **b** The effect of hyperosmotic stress on NF-κB activation in human aortic endothelial cells. Electrophoretic mobility gel shift assay (*EMSA*), showing the effect of hypertonic stress on NF-κB activation. EMSA was performed by mixing the ^32^P-oligonucleotide encoding for the NF-κB binding probe with nuclear extracts from osmotically stressed HAECs for 1–3 h. The electrophoretic run with nuclear protein extracts from unstimulated HAECs is shown in *lane* 1 and *lane* 7. The supershift analysis with a polyclonal rabbit antibody against p65, shows upward shift of binding complexes. Results are representative of three separate experiments. **c** Scanning densitometry of the shift bands (highlighted within the *rectangle*) of EMSA gels, expressed as arbitrary units of optical density. Data represent for each condition the mean ± S.D. from 3 separate experiments. *, P < 0.01 vs control HAECs

COX-2 is involved in human angiogenesis, although with mechanisms still incompletely understood [29]. To explore the involvement of hyperosmolarity in COX-2-induced angiogenesis, we seeded cells on Matrigel after incubation with high glucose or high mannitol in the presence or absence of siRNA against AQP1 or the COX-2 inhibitor NS-398. In this functional assay of angiogenesis, HAECs were treated with endothelial growth factor (EGF) as positive control for tubulization. EGF did induce tubule formation, and high glucose or high mannitol produced the highest number of network formations. Treatment with siRNA against AQP1 strongly reduced the number of these structures (Fig. 3A, C), while NS-398 reduced them to a lesser extent (Fig. 3B**).** TonEBP, also called nuclear factor of activated T cells (NFAT)-5, is a master transcription factor of genes responsive to hypertonic stress [30–32], TonEBP/NFAT5 siRNA did not affect cell morphology or cell proliferation (Fig. 3D), but triggered a substantial (80 %) reduction in TonEBP/NFAT5 protein expression (Fig. 3D). By contrast, serving as a sequence-unrelated control, scrambled siRNA did not exert any detectable effect. TonEBP/NFAT5 silencing drastically decreased the network number and area formed by HAECs seeded in Matrigel, compared with those obtained from glucose and mannitol-treated cells (Fig. 3E). Cell migration induced by high glucose or high mannitol in a modified Boyden chamber system was also quantified with a fluorimetric assay. Migration of TonEBP/NFAT5-deficient endothelial cells towards high glucose and high mannitol was significantly inhibited (Fig. 3F), indicating a role of TonEBP/NFAT5 in mediating hyperosmolarity-induced endothelial migration and angiogenesis. TonEBP/NFAT5 expression and binding activity was increased in cells exposed to high glucose and mannitol (Fig. 4a–d), and siRNA against TonEBP/NFAT5 24 h prevented the hypertonic induction of TonEBP/NFAT5 (Fig. 4a) and COX-2 proteins (Fig. 5a), indicating that TonEBP/NFAT5 expression and activity are required for the hyperosmolarity induction of COX-2. Taken together, these results showed that hyperosmolarity contributes to the stimulatory effect of COX-2 on endothelial angiogenesis mostly through the activation of upstream and downstream mediators of hyperosmolarity, such as AQP1 and TonEBP/NFAT5, respectively.

The promoter region of the COX-2 gene also contains consensus sequences for NF-κB [33–36], and high glucose induces the expression of some genes also by activating NF-κB [33, 37]. In order to analyze the contribution of NF-κB to the hyperosmotic stress-induced expression of COX-2, we performed EMSA using NF-κB binding probe and nuclear extracts from osmotically stressed HAECs for 1–24 h. We found low, but detectable amounts of NF-κB DNA binding activities in unstimulated cells. After treatment with high glucose (1 and 3 h) and TNF-α (up to 24 h), but not with high mannitol or sodium chloride, we observed a time-dependent increase in NF-κB DNA-binding activity, suggesting that this transcription factor can cooperate to the expression of the COX-2 gene by high glucose, but not in response to high glucose-related hyperosmotic stress (Fig. 5b–c).

### Retinas of D2.B6-*Ins2 Akita* diabetic mice exhibit high levels of hyperosmolarity-enhanced COX-2 expression and angiogenesis

**Fig. 6:**
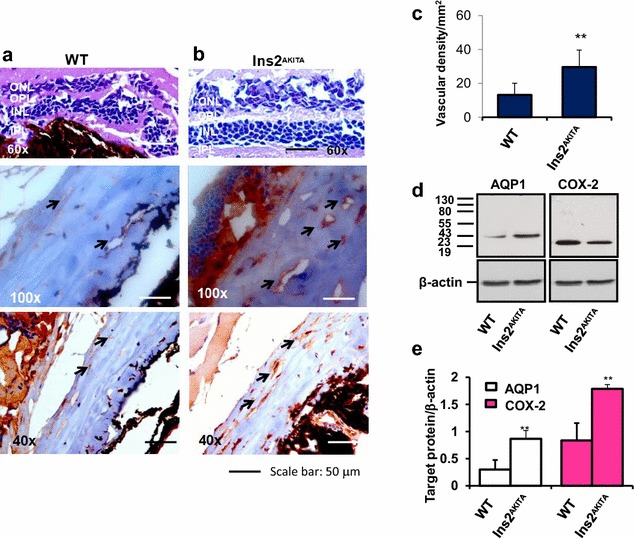
Retinal neovascularization and higher expression of AQP1 and COX-2 in the *Ins2Akita* mice. **a, b** Upper panels: Hematoxylin/eosin-stained retinal cross-sections from sex- and age-matched (1 year-old) C57BL/6 non-diabetic control mice (**a**) and D2.B6-*Ins2 Akita* (**b**). Magnification: 60x.* Lower panels*: immunohistochemistry of CD31-stained retinal cross-sections from D2.B6-*Ins2 Akita* (**a**) and sex- and age-matched (1 year-old) C57BL/6 non-diabetic control mice (**b**), showing higher CD31 immunoreactivity in the D2.B6-*Ins2 Akita* mice. Magnification: 40x and 100x. **c** The extent of angiogenesis was determined by measuring total vessels (arterioles and capillaries) density in light microscopy sections. N = 2 eyes per animal, with each experimental group consisting of n = 7 mice. The* bar graph* represents for each value the mean ± S.D. from 3 separate experiments. **, P < 0.01 *vs* C57BL/6 control mice. **d, e**: Western analysis of COX-2 and AQP1 expression in retinas from D2.B6-*Ins2 Akita* mouse and from sex- and age-matched (1 year-old) C57BL/6 non-diabetic control mice, with β-actin serving as a loading control. N = 2 eyes/group, with each group consisting of n = 7 mice. The *bar graph* represents for each value the mean ± S.D. from three separate experiments. **, P < 0.01 *vs* C57BL/6 control mice

**Fig. 7:**
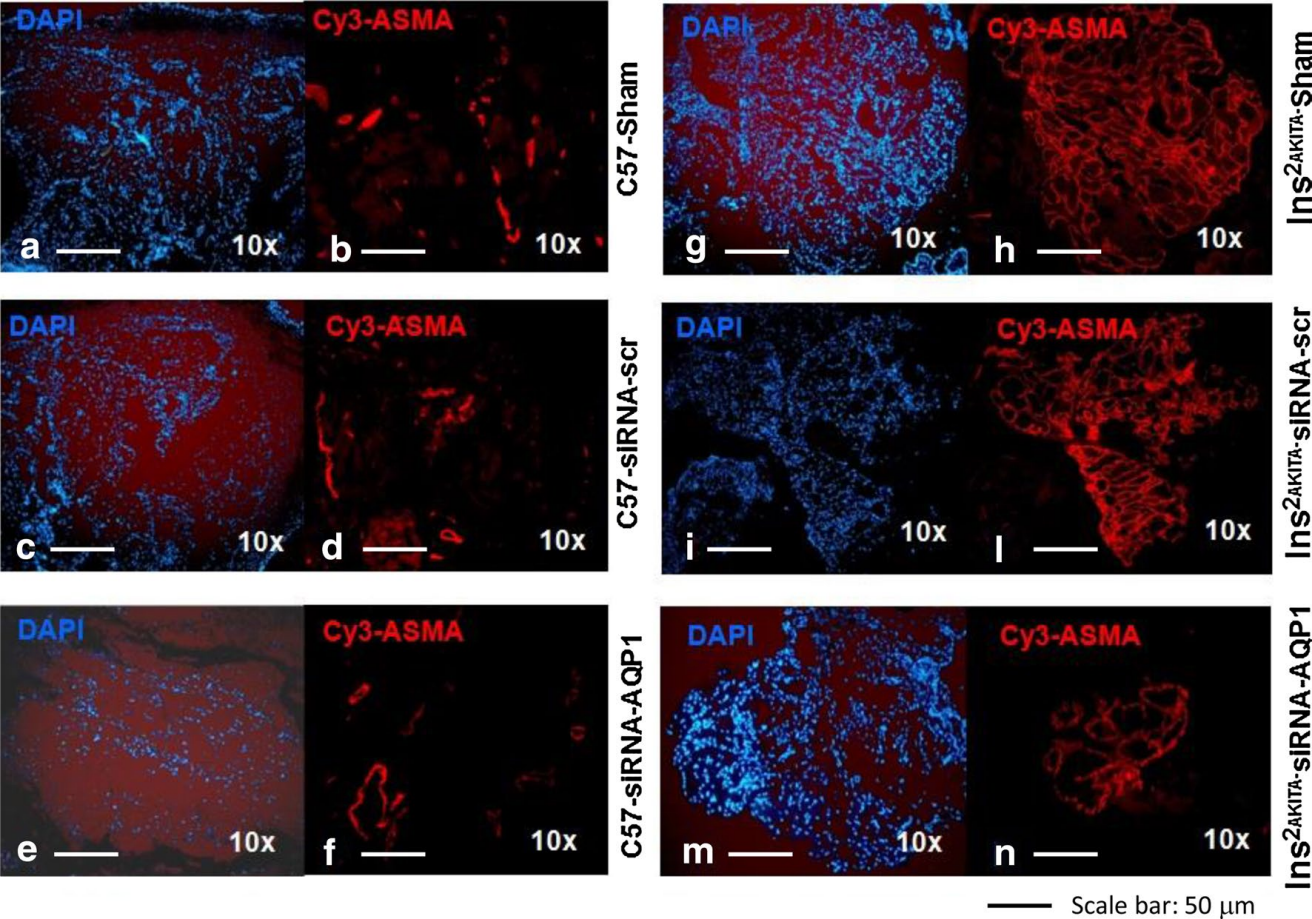
The involvement of AQP1 in retinal neovascularization in the *Ins2Akita* and C57BL/6 mice. Immunofluorescence staining of retinal cross-sections for α-smooth muscle actin (ASMA) and 4′,6-diamidino-2-phenylindole (DAPI) for nuclei from D2.B6-*Ins2 Akita* (G-N) and sex- and age-matched (1 year-old) C57BL/6 non-diabetic control mice (**a–f**), after intravitreal injection of saline (sham,* panels* **a, b g, h**), or siRNA with scrambled sequence (siRNA-scr,* panels* **c, d, i, l**) or siRNA against AQP1 (siRNA-AQP1,* panels* **e, f, n, m**). Magnification: 10x

**Fig. 8:**
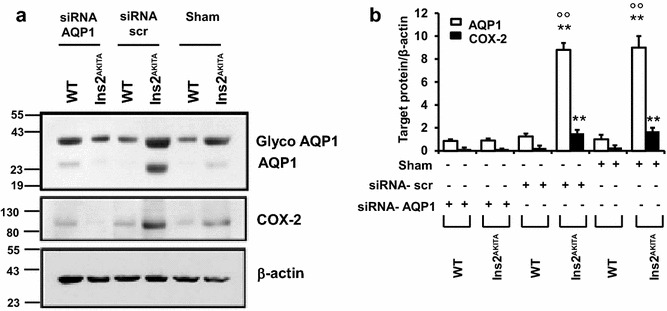
The involvement of AQP1 in COX-2 expression in the *Ins2Akita* and C57BL/6 mice. **a** Western analysis of COX-2 and AQP1 expression in retinas from from D2.B6-*Ins2 Akita* and sex- and age-matched (1 year-old) C57BL/6 non-diabetic control mice, after the intravitreal injection of saline (sham), or siRNA against scrambled sequence (siRNA-scr) or siRNA against AQP1 (siRNA-AQP1), with β-actin serving as a loading control. **b** n = 2 eyes per animal, with each experimental group consisting of n = 7 mice. The* bar graph* represents the means ± S.D. from three separate experiments. **, P < 0.01 vs C57BL/6 control mice °°, P < 0.01 vs sham and siRNA-scr. Glyco AQP1 represents the glycosylated form of AQP1 able to insert itself in the plasma membrane

CD31 (a marker of angiogenesis) and α-smooth muscle actin (ASMA, a marker of arteriogenesis), along with COX-2 and AQP1 were examined in retinas of D2.B6-*Ins2 Akita* diabetic mice and sex-age matched C57BL/6 non-diabetic controls treated with or without siRNAs to AQP1. We detected low-level CD31 (Fig. 6a) and ASMA immunoreactivities (Fig. 7a–d) in WT mice. However, a significant vessel staining for CD31 (Fig. 6b) and ASMA (Fig. 7g–l) were apparent in retinas of D2.B6-*Ins2 Akita* mice, mostly in the inner plexiform layer (IPL) and Outer plexiform layer (OPL). Retinas in diabetic mice also showed increased expression of AQP1 and a significant upregulation of COX-2 at the protein level (Fig. 6d–e). AQP1 in vivo silencing drastically decreased the number of ASMA-positive vascular structures (Fig. 7e–f, m–n) and the expression of AQP1 and COX-2 (Fig. 8a, b), in D2.B6-*Ins2 Akita* mice.

## Discussion

Clinical consequences of type-1 diabetes result from the combination of a number of different factors, ranging from acidosis to metabolic changes, to biophysical derangements, such as hyperosmotic stress. Previous research has mostly focused on metabolic disorders induced by hyperglycemia and the underlying signaling pathways. Several lines of evidence presented here however indicate that a substantial contribution to the induction of vascular disease is given by the hyperosmolar stress related to hyperglycemia. We show here that concentrations of glucose attainable in diabetes induce COX-2 in human endothelial cells through an AQP1-dependent hyperosmolar mechanism and, through this mechanism, promote angiogenesis. Specifically, in diabetic *Ins2Akita* mice, retinal COX-2 and AQP1 are upregulated concomitant with increased angiogenesis, suggesting the relevance of our observations in an in vivo setting where hyperglycemia is the main driver of microvascular disease. Finally, hyperosmolarity, through an AQP1-dependent mechanism appears to be causally involved in angiogenesis in vivo, and interruption of the AQP1 axis mitigates in vivo increased angiogenesis in response to high glucose.

This report expands our previous findings that hyperosmotic stress induces endothelial ICAM-1 and VCAM-1 expression [11] by showing the involvement of COX-2-dependent pathological phenomena. The current findings provide evidence that hyperosmolarity is a biophysical mechanism through which excessive angiogenesis may occur in diabetes. Additionally, our findings detail part of the molecular signaling through which hyperosmolarity promotes such effects.

The involvement of AQP1 in hyperosmolarity-induced endothelial tubulization and angiogenesis [38–41] is of biological importance. AQP1 is a water channel protein widely expressed in vascular endothelial cells, which, when activated, increases cell membrane water permeability [42–44]. AQP1 protein is strongly expressed in human and animal models of highly proliferating microvessels [43] and the chick embryo chorioallantoic membrane [44]. In human retinal epithelial cells, osmotic stress decreases AQP4 expression [45]. Reduction in AQP1 expression by targeted siRNA inhibits hyperosmolarity-dependent angiogenesis. In general, angiogenesis requires endothelial cell proliferation, adhesion and migration. We found a significant decrease in network number and areas in AQP1-silenced cultures, indicating a lost capacity to organize tubuli in networks. This is in line with previous reports showing that AQP1 plays a key role in endothelial cell migration, an early fundamental step in angiogenesis [10, 46].

Data from the present study also provide a mechanistic explanation on how AQP1 is linked to endothelial cell migration and tubulization. We observed that high glucose, at levels of 30.5 mmol/L, corresponding to 450 mg/dL, a plasma level possibly found in uncontrolled diabetes—increases COX-2 expression. This effect is in part dependent on the hyperosmolar component of hyperglycemia, since mimicked by the exposure of cells to the metabolically inactive monosaccharides mannitol or l-glucose.

TonEBP/NFAT5, a rel/NF-κB family member, has been identified as a transcription factor that in response to hyperosmolarity activates the transcription of osmosensing genes, including aldose reductase and protein kinase C delta [47], TNF-α and monocyte chemoattractant protein (MCP)-1 [30, 34–36]. In renal medullary epithelial cells, hypertonic stress increased COX-2 promoter activity in a luciferase reporter assay [37, 48]. Also, hypertonic saline had been reported to induce COX-2 in rat macrophages [49] and HUVECs [50], but in experimental conditions that do not reproduce the diabetic milieu. The participation of NFAT transcription factors in the activation of COX-2 expression had also been already demonstrated in several cell types [31, 32, 51–54]. Indeed, previous research had shown that the angiogenic signaling involved in the induction of COX-2 by vascular endothelial growth factor (VEGF) in human endothelial cells requires the activation of NFAT proteins [51, 55]. Furthermore, TonEBP/NFAT5 is required for the hypertonic induction of COX-2 in renal epithelial cells [31]. An upregulation of the NFAT-MMP-2 pathway had been also demonstrated in the development of metastatic osteosarcoma, where the secretion of active MMP-2 is under the transcriptional regulation of NFAT [32]. Here however we provide the first evidence that the induction of COX-2 in endothelial cells is largely hyperosmolarity-related, and that TonEBP/NFAT5 is here involved, since (a) TonEBP/NFAT total abundance was increased in cells exposed to high glucose or high mannitol; (b) EMSA and Western analysis demonstrated a cytoplasm-to-nucleus redistribution of TonEBP/NFAT in endothelial cells; and (c) TonEBP/NFAT gene disruption prevented hyperosmolarity-related induction of COX-2. Of note, our data show that at least another signal transduction pathways activated by high glucose, but with lesser relevance to COX-2 expression and angiogenesis, such as the NF-κB pathway, is—conversely—not substantially induced by high mannitol, indicating a larger dependence of NF-κB activation on metabolic effects of glucose.

A number of studies have shown that hyperosmolarity (as tested by high mannitol) does not influence angiogenesis and/or COX-2 expression in endothelial cells [55, 56]. Culture conditions used in these studies may not reflect those used in our study. Our data show more robust effects when using glucose concentrations as high as 50.5 mM, or with lower concentrations but for prolonged times, which may more closely mimic diabetic pathophysiology. Concentrations of glucose outside the range tested here might exert different effects, therefore not completely ruling out the additional involvement of mechanisms different from hyperosmolarity. Nevertheless, our data clearly indicate that hyperosmolarity is an important part of the mechanisms through which chronic exposure to high glucose may promote angiogenesis and cardiovascular complications of diabetes.

We recognize some limitations of our study. Main ones are that (a) there is limited translability of murine data to humans; (b) molecular mechanisms by which TonEBP/NFAT operates on the COX-2 promoter are still incompletely understood; (c) we still need to close the circle of the in vivo causality demonstration involving the transcription factor TonEBP/NFAT5 as an additional osmosignaling effector by showing that the in vivo interruption of the TonEBP signaling mitigates the in vivo increased angiogenesis in response to high glucose. Such experiments are complicated by the lack of available transgenic mouse models of TonEBP knockouts. Such limitations might however be overcome in the future through the prolonged administration of drugs targeting TonEBP activation.

**Fig. 9:**
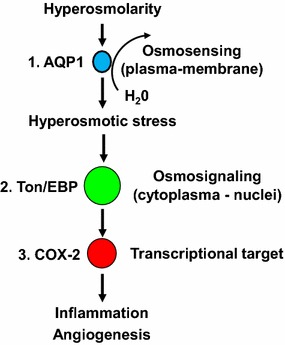
A scheme of the putative signaling pathway through which hyperosmotic stress contributes to some of the vascular events in diabetes, based on findings from the present study. In the plasma membrane, hyperglycemia activates “osmosensing” structures, such as aquaporin (AQP)-1, which can sense osmolarity changes; and an “osmosignaling” pathway, involving the transcription factor tonicity enhancer binding protein (Ton/EBP), which can transmit the signal towards effector regulatory sites in the nuclei, able to further promote the expression of proinflammatory genes such as adhesion molecules and cycloxygenase (COX)-2

Nonetheless, data provided here lend support to the hypothesis that hyperosmolarity is an important mechanism of vascular disease in diabetes and a potential target for future therapies, with several potential practical implications. In principle, promotion of inflammation and angiogenesis by COX-2 through a hyperosmolarity-related mechanism may have a role not only in microvascular disease (proliferative retinopathy), as shown here, but also in macrovascular disease (e.g., plaque rupture), both increased in diabetes. The unraveling of the hyperosmolarity-related signaling, as sketched in Fig. 9, pointing both to the sensing molecule AQP1 and the effector molecule COX-2, allows identifying potential pharmacological targets to reduce hyperosmolar stress-related excessive angiogenesis. In addition, the in vivo expressions of AQP1 and COX-2 might serve as new markers for diabetic complications. The in vivo implications of these observations are therefore worth further assessment.

## References

[1] UK Prospective Diabetes Study UKPDS) Group (1998). Effect of intensive blood-glucose control with metformin on complications in overweight patients with type 2 diabetes (UKPDS 34. Lancet.

[2] Holman RR, Paul SK, Bethel MA, Matthews DR, Neil HA (2008). 10-year follow-up of intensive glucose control in type 2 diabetes. N Engl J Med.

[3] Brownlee M (2001). Biochemistry and molecular cell biology of diabetic complications. Nature.

[4] Paget C, Lecomte M, Ruggiero D, Wiernsperger N, Lagarde M (1998). Modification of enzymatic antioxidants in retinal microvascular cells by glucose or advanced glycation end products. Free Radic Biol Med.

[5] Basta G, Lazzerini G, Massaro M, Simoncini T, Tanganelli P, Fu C, Kislinger T, Stern DM, Schmidt AM, De Caterina R (2002). Advanced glycation end products activate endothelium through signal-transduction receptor RAGE: a mechanism for amplification of inflammatory responses. Circulation.

[6] Ishii H, Koya D, King GL (1998). Protein kinase C activation and its role in the development of vascular complications in diabetes mellitus. J Mol Med.

[7] Shanmugam N, Gaw Gonzalo IT, Natarajan R (2004). Molecular mechanisms of high glucose-induced cyclooxygenase-2 expression in monocytes. Diabetes.

[8] Madonna R, De Caterina R (2011). Cellular and molecular mechanisms of vascular injury in diabetes–part I: pathways of vascular disease in diabetes. Vascul Pharmacol.

[9] Zhang W, Liu H, Al-Shabrawey M, Caldwell RW, Caldwell RB (2011). Inflammation and diabetic retinal microvascular complications. J Cardiovasc Dis Res.

[10] Moreno PR, Fuster V (2004). New aspects in the pathogenesis of diabetic atherothrombosis. J Am Coll Cardiol.

[11] Madonna R, Montebello E, Lazzerini G, Zurro M, De Caterina R (2010). NA +/H + exchanger 1- and aquaporin-1-dependent hyperosmolarity changes decrease nitric oxide production and induce VCAM-1 expression in endothelial cells exposed to high glucose. Int J Immunopathol Pharmacol.

[12] Dubois RN, Abramson SB, Crofford L, Gupta RA, Simon LS, Van De Putte LB, Lipsky PE (1998). Cyclooxygenase in biology and disease. FASEB J.

[13] Tsujii M, Kawano S, Tsuji S, Sawaoka H, Hori M, DuBois RN (1998). Cyclooxygenase regulates angiogenesis induced by colon cancer cells. Cell.

[14] Masferrer JL, Leahy KM, Koki AT, Zweifel BS, Settle SL, Woerner BM, Edwards DA, Flickinger AG, Moore RJ, Seibert K (2000). Antiangiogenic and antitumor activities of cyclooxygenase-2 inhibitors. Cancer Res.

[15] Amano H, Ito Y, Suzuki T, Kato S, Matsui Y, Ogawa F, Murata T, Sugimoto Y, Senior R, Kitasato H (2009). Roles of a prostaglandin E-type receptor, EP3, in upregulation of matrix metalloproteinase-9 and vascular endothelial growth factor during enhancement of tumor metastasis. Cancer Sci.

[16] Conway EM, Collen D, Carmeliet P (2001). Molecular mechanisms of blood vessel growth. Cardiovasc Res.

[17] Toomey DP, Murphy JF, Conlon KC (2009). COX-2, VEGF and tumour angiogenesis. Surgeon.

[18] Frank RN (2004). Diabetic retinopathy. N Engl J Med.

[19] Carmeliet P (2003). Angiogenesis in health and disease. Nat Med.

[20] Roy S, Kim D, Hernandez C, Simo R (2015). Beneficial effects of fenofibric acid on overexpression of extracellular matrix components, COX-2, and impairment of endothelial permeability associated with diabetic retinopathy. Exp Eye Res.

[21] Peeters SA, Engelen L, Buijs J, Chaturvedi N, Fuller JH, Schalkwijk CG, Stehouwer CD (2015). Plasma levels of matrix metalloproteinase-2, -3, -10, and tissue inhibitor of metalloproteinase-1 are associated with vascular complications in patients with type 1 diabetes: the EURODIAB Prospective Complications Study. Cardiovasc Diabetol.

[22] Cosentino F, Eto M, De Paolis P, van der Loo B, Bachschmid M, Ullrich V, Kouroedov A, Delli Gatti C, Joch H, Volpe M (2003). High glucose causes upregulation of cyclooxygenase-2 and alters prostanoid profile in human endothelial cells: role of protein kinase C and reactive oxygen species. Circulation.

[23] Barber AJ, Antonetti DA, Kern TS, Reiter CE, Soans RS, Krady JK, Levison SW, Gardner TW, Bronson SK (2005). The Ins2Akita mouse as a model of early retinal complications in diabetes. Invest Ophthalmol Vis Sci.

[24] Han Z, Guo J, Conley SM, Naash MI (2013). Retinal angiogenesis in the Ins2(Akita) mouse model of diabetic retinopathy. Invest Ophthalmol Vis Sci.

[25] Madonna R, Di Napoli P, Massaro M, Grilli A, Felaco M, De Caterina A, Tang D, De Caterina R, Geng YJ (2005). Simvastatin attenuates expression of cytokine-inducible nitric-oxide synthase in embryonic cardiac myoblasts. J Biol Chem.

[26] Madonna R, Geng YJ, Shelat H, Ferdinandy P, De Caterina R (2014). High glucose-induced hyperosmolarity impacts proliferation, cytoskeleton remodeling and migration of human induced pluripotent stem cells via aquaporin-1. Biochim Biophys Acta.

[27] Turchinovich A, Zoidl G, Dermietzel R (2010). Non-viral siRNA delivery into the mouse retina in vivo. BMC Ophthalmol.

[28] Lang F, Busch GL, Ritter M, Volkl H, Waldegger S, Gulbins E, Haussinger D (1998). Functional significance of cell volume regulatory mechanisms. Physiol Rev.

[29] Gately S, Li WW (2004). Multiple roles of COX-2 in tumor angiogenesis: a target for antiangiogenic therapy. Semin Oncol.

[30] Esensten JH, Tsytsykova AV, Lopez-Rodriguez C, Ligeiro FA, Rao A, Goldfeld AE (2005). NFAT5 binds to the TNF promoter distinctly from NFATp, c, 3 and 4, and activates TNF transcription during hypertonic stress alone. Nucleic Acids Res.

[31] Favale NO, Casali CI, Lepera LG, Pescio LG, Fernandez-Tome MC (2009). Hypertonic induction of COX2 expression requires TonEBP/NFAT5 in renal epithelial cells. Biochem Biophys Res Commun.

[32] Velupillai P, Sung CK, Tian Y, Dahl J, Carroll J, Bronson R, Benjamin T (2010). Polyoma virus-induced osteosarcomas in inbred strains of mice: host determinants of metastasis. PLoS Pathog.

[33] Chen G, Shen X, Yao J, Chen F, Lin X, Qiao Y, You T, Lin F, Fang X, Zou X (2009). Ablation of NF-kappaB expression by small interference RNA prevents the dysfunction of human umbilical vein endothelial cells induced by high glucose. Endocrine.

[34] Miyakawa H, Woo SK, Chen CP, Dahl SC, Handler JS, Kwon HM (1998). Cis- and trans-acting factors regulating transcription of the BGT1 gene in response to hypertonicity. Am J Physiol.

[35] Takenaka M, Preston AS, Kwon HM, Handler JS (1994). The tonicity-sensitive element that mediates increased transcription of the betaine transporter gene in response to hypertonic stress. J Biol Chem.

[36] Kojima R, Taniguchi H, Tsuzuki A, Nakamura K, Sakakura Y, Ito M (2010). Hypertonicity-induced expression of monocyte chemoattractant protein-1 through a novel cis-acting element and MAPK signaling pathways. J Immunol.

[37] Zhao H, Tian W, Tai C, Cohen DM (2003). Hypertonic induction of COX-2 expression in renal medullary epithelial cells requires transactivation of the EGFR. Am J Physiol Renal Physiol.

[38] Kaneko K, Yagui K, Tanaka A, Yoshihara K, Ishikawa K, Takahashi K, Bujo H, Sakurai K, Saito Y (2008). Aquaporin 1 is required for hypoxia-inducible angiogenesis in human retinal vascular endothelial cells. Microvasc Res.

[39] Saadoun S, Papadopoulos MC, Hara-Chikuma M, Verkman AS (2005). Impairment of angiogenesis and cell migration by targeted aquaporin-1 gene disruption. Nature.

[40] Maruyama T, Kadowaki H, Okamoto N, Nagai A, Naguro I, Matsuzawa A, Shibuya H, Tanaka K, Murata S, Takeda K (2010). CHIP-dependent termination of MEKK2 regulates temporal ERK activation required for proper hyperosmotic response. EMBO J.

[41] Umenishi F, Schrier RW (2003). Hypertonicity-induced aquaporin-1 (AQP1) expression is mediated by the activation of MAPK pathways and hypertonicity-responsive element in the AQP1 gene. J Biol Chem.

[42] Carter EP, Olveczky BP, Matthay MA, Verkman AS (1998). High microvascular endothelial water permeability in mouse lung measured by a pleural surface fluorescence method. Biophys J.

[43] Saadoun S, Papadopoulos MC, Davies DC, Bell BA, Krishna S (2002). Increased aquaporin 1 water channel expression in human brain tumours. Br J Cancer.

[44] Ribatti D, Frigeri A, Nico B, Nicchia GP, De Giorgis M, Roncali L, Svelto M (2002). Aquaporin-1 expression in the chick embryo chorioallantoic membrane. Anat Rec.

[45] Willermain F, Janssens S, Arsenijevic T, Piens I, Bolaky N, Caspers L, Perret J, Delporte C (2014). Osmotic stress decreases aquaporin-4 expression in the human retinal pigment epithelial cell line, ARPE-19. Int J Mol Med.

[46] Clapp C, de la Escalera GM (2006). Aquaporin-1: a novel promoter of tumor angiogenesis. Trends Endocrinol Metab.

[47] Park J, Kim H, Park SY, Lim SW, Kim YS, Lee DH, Roh GS, Kim HJ, Kang SS, Cho GJ (2014). Tonicity-responsive enhancer binding protein regulates the expression of aldose reductase and protein kinase C delta in a mouse model of diabetic retinopathy. Exp Eye Res.

[48] Pucci ML, Endo S, Nomura T, Lu R, Khine C, Chan BS, Bao Y, Schuster VL (2006). Coordinate control of prostaglandin E2 synthesis and uptake by hyperosmolarity in renal medullary interstitial cells. Am J Physiol Renal Physiol.

[49] Zhang W, Zitron E, Homme M, Kihm L, Morath C, Scherer D, Hegge S, Thomas D, Schmitt CP, Zeier M (2007). Aquaporin-1 channel function is positively regulated by protein kinase C. J Biol Chem.

[50] Arbabi S, Rosengart MR, Garcia I, Maier RV (2000). Hypertonic saline solution induces prostacyclin production by increasing cyclooxygenase-2 expression. Surgery.

[51] Hernandez GL, Volpert OV, Iniguez MA, Lorenzo E, Martinez-Martinez S, Grau R, Fresno M, Redondo JM (2001). Selective inhibition of vascular endothelial growth factor-mediated angiogenesis by cyclosporin A: roles of the nuclear factor of activated T cells and cyclooxygenase 2. J Exp Med.

[52] Duque J, Fresno M, Iniguez MA (2005). Expression and function of the nuclear factor of activated T cells in colon carcinoma cells: involvement in the regulation of cyclooxygenase-2. J Biol Chem.

[53] Yiu GK, Toker A (2006). NFAT induces breast cancer cell invasion by promoting the induction of cyclooxygenase-2. J Biol Chem.

[54] Yan Y, Li J, Ouyang W, Ma Q, Hu Y, Zhang D, Ding J, Qu Q, Subbaramaiah K, Huang C (2006). NFAT3 is specifically required for TNF-alpha-induced cyclooxygenase-2 (COX-2) expression and transformation of Cl41 cells. J Cell Sci.

[55] Giurdanella G, Anfuso CD, Olivieri M, Lupo G, Caporarello N, Eandi CM, Drago F, Bucolo C, Salomone S (2015). Aflibercept, bevacizumab and ranibizumab prevent glucose-induced damage in human retinal pericytes in vitro, through a PLA2/COX-2/VEGF-A pathway. Biochem Pharmacol.

[56] Lupo G, Motta C, Giurdanella G, Anfuso CD, Alberghina M, Drago F, Salomone S, Bucolo C (2013). Role of phospholipases A2 in diabetic retinopathy: in vitro and in vivo studies. Biochem Pharmacol.

